# Multitargeting Pt(IV) Anticancer Prodrugs Bearing Mono- and Bis-Probenecid Ligands in Axial Positions: Synthesis and Evaluation of Biological Activity

**DOI:** 10.3390/ph19030386

**Published:** 2026-02-27

**Authors:** Panxing Qiu, Yu Zhang, Yang Dou, Zhijin Cheng, Xiaoqin Wu, Silong Zhang, Fuyi Wang, Kui Wu

**Affiliations:** 1 Key Laboratory of Hubei Province for Coal Conversion and New Carbon Materials, School of Chemistry and Chemical Engineering, Wuhan University of Science and Technology, Wuhan 430081, Chinac13561096366@163.com (Z.C.); wuxiaoqin@wust.edu.cn (X.W.); 2 School of Pharmaceutical Sciences, Guizhou University, Guiyang 550025, Chinasilongzhang@whu.edu.cn (S.Z.); 3 Beijing National Laboratory for Molecular Sciences, National Centre for Mass Spectrometry in Beijing, CAS Key Laboratory of Analytical Chemistry for Living Biosystems, Institute of Chemistry, Chinese Academy of Sciences, Beijing 100190, China; douyang@iccas.ac.cn; 4 University of Chinese Academy of Sciences, Beijing 100049, China

**Keywords:** Pt(IV) prodrug, probenecid, anticancer, metallodrug, multitargeting

## Abstract

**Background**: To battle the side effects of anticancer Pt(II) drug cisplatin, the development of photoactivatable and/or intracellular reduction-activatable Pt(IV) prodrugs has become a promising strategy. **Methods:** Herein, two novel Pt(IV) prodrugs, namely, *cis*,*cis*,*trans*-[Pt^IV^(NH_3_)_2_(Cl)_2_(OH)(probenecid)]) (SPP) and *cis*,*cis*,*trans*-[Pt^IV^(NH_3_)_2_(Cl)_2_(probenecid)_2_] (DPP) bearing mono- and di-probenecid at the axial positions of oxoplatin have been synthesized via covalently linking of carboxylate group in probenecid, which is a well-established clinic drug by inhibiting organic anion transporter 1 (OAT1) to reduce cisplatin-induced nephrotoxicity, with the axial hydroxyl group(s) in oxoplatin. The promising cytotoxicity of SPP and DPP against MCF-7, T47D breast cancer cells and the MDA-MB-231 triple-negative breast cancer cells was evaluated, and the mechanism of action of the two Pt(IV) prodrugs was investigated by apoptosis assay and Western blot assay. **Results**: SPP exhibits a comparable cytotoxicity to cisplatin against MCF-7 and T47D breast cancer cells, while it shows 2.1-fold higher cytotoxicity than cisplatin against MDA-MB-231 cells. DPP was shown to be more cytotoxic than SPP, and exhibits 8.7-, 7.5-, and 2.3-fold higher cytotoxicity than cisplatin against MCF-7, T47D, and MDA-MB-231 cells, respectively. Apoptosis assays revealed a similar early-apoptotic cell death mechanism to cisplatin for both SPP and DPP. The enhanced cellular and nuclear uptake of DPP compared to cisplatin contributes to its promising anticancer efficacy. DPP can bind to OAT1 in cancer cells, which may synergistically enhance the cytotoxicity of the Pt(IV) anticancer prodrugs. **Conclusions**: The direct conjugation of probenecid to the axial positions of oxoplatin confers the resulting Pt(IV) prodrugs a multitargeting property, significantly promoting the cytotoxicity of the resulting Pt(IV) complexes. This finding provides a practical strategy for drug design and cancer treatment based on platinum complexes.

## 1. Introduction

Platinum(II) drugs, e.g., cisplatin (CDDP), carboplatin, and oxaliplatin, are front-line chemotherapeutic agents for the treatment of solid tumors, including testicular, breast, and lung cancers [1,2,3,4,5,6]. However, platinum(II) drugs face many problems in the clinic, such as poor tumor selectivity, non-specific toxicity (e.g., nephrotoxicity, neurotoxicity), and inherent or acquired drug resistance [6,7]. To address these limitations, research efforts have shifted towards non-classical platinum complexes with novel mechanisms of action [8,9], for instance, platinum(IV) prodrugs. Unlike square-planar Pt(II) complexes, Pt(IV) prodrugs adopt an octahedral configuration, rendering them kinetically inert under physiological conditions. This stability prevents ligand substitution in physiological environments, thereby reducing non-specific activation of the drugs and minimizing off-target toxicity before they reach tumors [5]. Moreover, the two axial sites in Pt(IV) prodrugs allow functionalization with bioactive ligands, enabling modulation of properties such as lipophilicity, redox stability, tumor targeting, and multitargeting functions. This can confer synergistic biological activities and enhance cellular uptake of platinum, providing a promising approach to overcoming the limitations of Pt(II)-based drugs [10,11,12,13,14,15].

Probenecid is a well-established drug to treat gout and reduce the renal excretion of antibiotics [16], and has recently gained much more attention due to its ability to interact with multiple membrane transporters and channel proteins, such as Transient Receptor Potential Vanilloid 2 (TRPV2), organic anion transporters (OAT), and pannexin 1 hemichannels [17]. Especially for the OAT family protein OAT1, in vitro studies in OAT1-knockout mice revealed significantly reduced renal damage by CDDP compared to wild-type mice, where a significant increase in creatinine, a marker of renal tubular necrosis, was observed. Probenecid was also shown to downregulate OAT expression and reduce the accumulation of uremic toxins induced by CDDP, in turn alleviating CDDP-induced nephrotoxicity [18]. Moreover, growing evidence suggests that probenecid is a potent modulator of activity that reverses the expression of multidrug resistance proteins (MRPs), increasing effective serum concentrations of chemotherapeutics [19,20,21,22,23,24]. Probenecid has also been demonstrated to inhibit glutathione conjugate export pumps, thereby enhancing the activity of Pt(II) drugs by blocking detoxification pathways [25,26,27]. These make probenecid an ideal axial ligand for Pt(IV) prodrug design, potentially conferring the resulting Pt(IV) complexes with both enhanced anticancer activity and reduced toxicity.

To this end, in the present work, we report the design and synthesis of novel mono- and di-probenecid conjugated Pt(IV) prodrugs, in which the carboxyl group of probenecid is directly linked to the axial hydroxyl groups in oxoplatin without additional modifications. The preliminary evaluation of biological activity for the resulting Pt(IV) complexes indicates that the conjugation of probenecid as the axial ligand(s) indeed promotes the cytotoxicity of the Pt(IV) against breast cancer cells, in particular against triple-negative breast cancer (TNBC) cells. The possible mechanism underlying the enhancement of the cytotoxicity of the Pt(IV) prodrugs was investigated by apoptosis assay and Western blot assay.

## 2. Results

### 2.1. Synthesis and Characterization

**Scheme 1:**
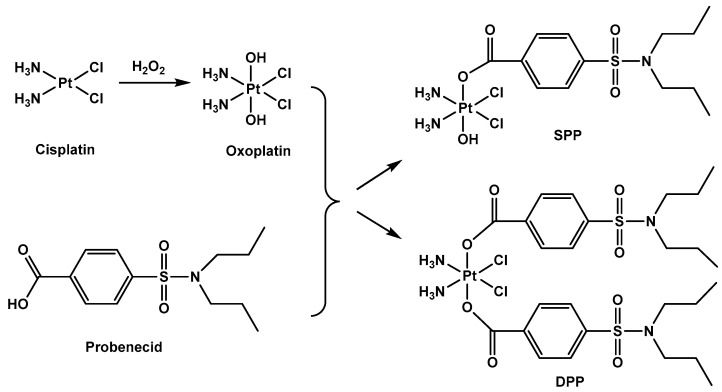
Synthetic route to prepare SPP and DPP in anhydrous DMF containing TBTU and TEA.

The precursor oxoplatin was prepared by oxidizing CDDP with 30% H_2_O_2_. The target Pt(IV) prodrugs, *cis*,*cis*,*trans*-[Pt^IV^(NH_3_)_2_(Cl)_2_(OH)(probenecid)] (SPP) and *cis*,*cis*,*trans*-[Pt^IV^(NH_3_)_2_(Cl)_2_(probenecid)_2_] (DPP), were synthesized via direct esterification of probenecid with oxoplatin in the presence of O-(benzotriazole-1-yl)-N,N,N′,N′-tetramethylurea tetrafluoroborate (TBTU) and triethylamine (TEA) in anhydrous N,N-dimethylformamide (DMF) (Scheme 1), where TBTU serves as a coupling agent and TEA as a base. The reaction conditions for DPP were optimized (55 °C, 24 h) to ensure bis-conjugation, while SPP was synthesized at 25 °C for 12 h. Silica gel column chromatography using a mixture of CH_2_Cl_2_:MeOH (5:1, *v*/*v*) as solvent afforded pure products with yields of 21% (SPP) and 51% (DPP), respectively. The structures and purity of SPP and DPP were fully characterized by ^1^H NMR, ^13^C NMR spectroscopy, and high-resolution mass spectrometry (Figures S1–S6 in the Supplementary Materials), which supported the proposed structures of the two complexes. In the ^1^H NMR spectra of SPP and DPP, amino groups showed resonance peaks at δ 5.8–6.1 ppm and 6.5–6.7 ppm, respectively, which were similar to those of other Pt(IV) complexes [4,28]. The structures of mono- and di-probenecid ligands were also confirmed by the isotopic distribution patterns and well-matched *m*/*z* for SPP at 602.06 ([M+H]^+^, calc. *m*/*z* 602.41) and DPP at 869.15 ([M+H]^+^, calc. *m*/*z* 869.75) in the mass spectrum.

### 2.2. Stability and Reduction

**Figure 1:**
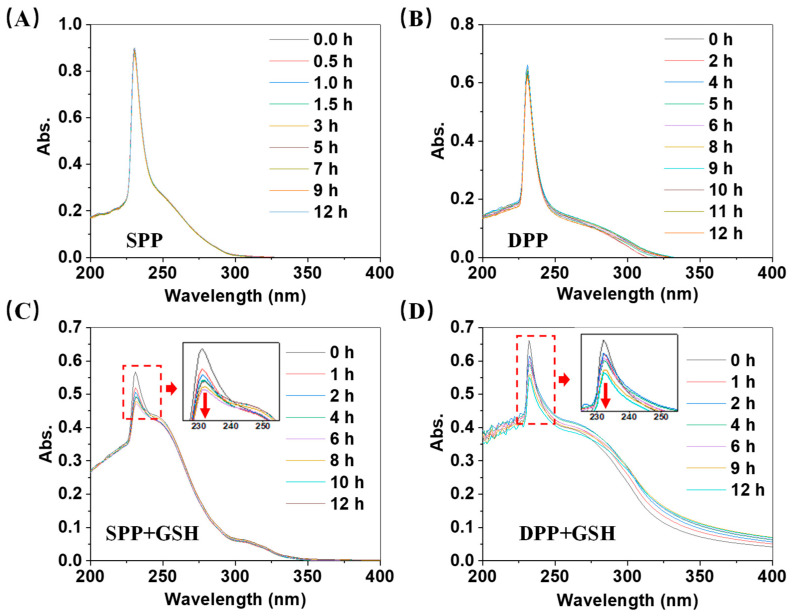
UV–vis spectra of SPP (**A**,**C**) and DPP (**B**,**D**) in PBS in the absence (**A**,**B**) or presence (**C**,**D**) of GSH (200 μM).

Stability in the bloodstream is crucial for the selective anti-tumor activity of Pt(IV) prodrugs, which prevent the reduction and activation of Pt(IV) in the bloodstream before the prodrugs enter cancer cells [29]. According to the well-accepted knowledge, Pt(IV) prodrugs would be reduced to various Pt(II) species by intracellular reductants and then form Pt(II)-DNA adducts within the nucleus to exert anti-tumor activity [30]. To mimic the intracellular environment, the redox stability of SPP and DPP was studied by incubating the complexes with intracellular reductant glutathione (GSH), and the reaction mixtures were monitored by UV–vis spectroscopy (Figure 1). SPP or DPP in PBS without GSH was used as a control. The results showed that SPP and DPP were stable in PBS during the 12 h incubation in the absence of GSH with no intensity change in their UV–vis absorption at ~220 nm. While incubated with GSH at a concentration of 200 μM, a much lower concentration than the intracellular concentration (~2 to 10 mM) of GSH, the absorbance intensities of the reaction mixtures at 220 nm gradually decreased with increasing incubation time. This clearly indicated that both the Pt(IV) complexes, SPP and DPP, can serve as reduction-activatable prodrugs to release probenecid and CDDP-like species.

### 2.3. In Vitro Anti-Proliferative Activities

The in vitro anti-proliferative activities of the target complexes SPP and DPP were assessed via CCK8 assay with CDDP as a positive control. After 72 h of incubation with the human breast cancer cell lines MCF-7, T47D, and the triple-negative breast cancer (TNBC) cell line MDA-MB-231, both SPP and DPP exhibited dose-dependent inhibition of the proliferation of the tested cancer cells (Figure S7). According to the measured IC_50_ listed in Table 1, SPP showed comparable cytotoxicity to CDDP against MCF-7 and T47D cells, and higher cytotoxicity than CDDP towards MDA-MB-231 cells. DPP exhibited higher anti-proliferative activity against all three cancer cell lines than CDDP. The IC_50_ value of DPP against MCF-7 cells was 0.61 ± 0.02 μM, which was nearly 6.8- and 8.7-fold lower than those of SPP (4.13 ± 0.11 μM) and CDDP (5.29 ± 0.15 μM), respectively, and against T47D cells, the IC_50_ values of DPP were 7.8-fold and 7.2-fold lower than those of SPP and CDDP, respectively. While for the TNBC MDA-MB-231 cells, both SPP and DPP exhibited stronger anti-proliferative activity than CDDP (Table 1). These results indicate that the introduction of probenecid ligands at the axial positions of oxoplatin endows the Pt(IV) complexes with enhanced anti-tumor activity towards breast cancer cells, in particular to the TNBC cells, compared to CDDP. The dual ligation with probenecid in DPP appears to enhance the anticancer activity of the Pt(IV) complexes against MCF-7 and T47D cells in comparison with SPP bearing a solo probenecid ligand. In contrast, similar dual ligation with a hydroxyl group did not bring improvements in oxoplatin compared to cisplatin against several cancer cell lines [31], which further confirms the significance of probenecid ligands.

### 2.4. Cellular Uptake

**Figure 2:**
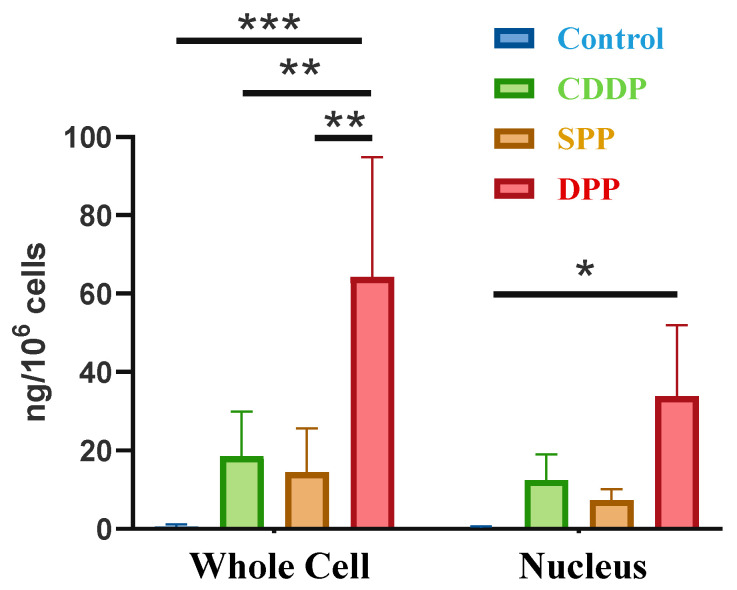
Cellular uptake of Pt in the whole cells or in the nuclei of MCF-7 cells after exposure to 20 μM SPP, DPP, or CDDP for 24 h. Results were shown as mean ± SD of three independent experiments. * *p* < 0.05, ** *p* < 0.01, *** *p* < 0.001.

To understand the mechanism of the enhanced anti-tumor activity of SPP and DPP against breast cancer cells, the intracellular uptake of SPP, DPP, and CDDP, indicated by the platinum level in MCF-7 cells incubated with 20 μM of the three complexes for 24 h at 37 °C, was measured by inductively coupled plasma mass spectrometry (ICP-MS). The results are shown in Figure 2. It can be seen that DPP had the highest cellular Pt accumulation both in the whole cells and in the nucleus, while SPP showed a slightly lower Pt accumulation than CDDP. The cellular uptake followed the order as DPP > CDDP ≈ SPP, which was in line with the cytotoxicity of them to MCF-7 cancer cells. The conjugation of a dual probenecid ligand within the Pt(IV) scaffold enhances the lipophilicity of the Pt(IV) complex, thereby promoting the cellular uptake of the complex, which may contribute to the elevated cytotoxicity of DPP. 

### 2.5. Apoptosis

**Figure 3:**
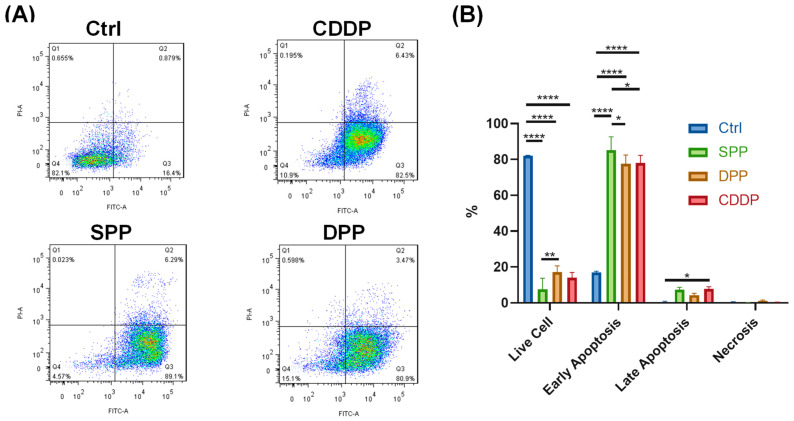
(**A**) Representative apoptosis assay results of tested complexes on MCF-7 cells by flow cytometry. Q1 represents the percentage of necrotic cells, Q2 late apoptotic cells, Q3 early apoptotic cells, and Q4 living cells. (**B**) Average Population of various statuses of cells based on triple independent experiments. * *p* < 0.05, ** *p* < 0.01 and **** *p* < 0.0001.

Apoptosis is one of the major cell death pathways induced by anti-tumor agents such as CDDP and carboplatin [3,32]. Therefore, to further investigate the anti-tumor mechanisms of SPP and DPP, apoptosis assays in MCF-7 cells were performed. MCF-7 cells were treated with 20 μM SPP, DPP, or CDDP for 24 h, stained with Annexin V-FITC/propidium iodide (PI) double dyer, and analyzed via flow cytometry. The representative cellular density plots (Figure 3A) and the corresponding average percentage of various statuses of cells based on three independent experiments (Figure 3B) are shown in Figure 3. The results demonstrated that both SPP and DPP mainly induced early apoptosis of MCF-7 cancer cells, with the rate as 85.1% and 77.5%, respectively. The late apoptosis rate was much lower than the early apoptosis rate. Such an early apoptosis mechanism was similar to that of CDDP (78.0%). SPP showed a slightly higher apoptotic rate than both DPP and CDDP. This suggested that the introduction of probenecid into Pt(IV) prodrugs did not change the action inducing cancer cell death of Pt(II)-based anticancer complexes such as CDDP. Probenecid might exert its positive effects through a non-apoptotic pathway.

### 2.6. Thermal Shift Binding Assay

**Figure 4:**
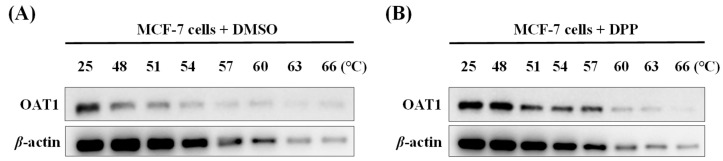
Thermal shift binding assay in the absence (**A**) or presence (**B**) of 100 μM DPP in MCF-7 cells to evaluate the interaction between OAT1 and DPP.

To verify whether the probenecid ligation confers the resulting Pt(IV) complexes binding affinity to the well-known target organic anion transporter 1 (OAT1) of probenecid, the binding interaction between DPP and OAT1 was investigated by thermal shift binding assay, a method that relies on the stabilization and protection of the target protein against denaturation and/or precipitation at elevated temperatures upon ligand binding [33]. Ligand binding to the protein induces an increase in its thermal stability. As evident in Figure 4A, in the DMSO group, increasing the temperature from 25 to 48 °C significantly diminished the band intensity of OAT1 expressed in MCF-7, and the band almost disappeared when the temperature was elevated to 54 °C. While in the MCF-7 cells treated with 100 μM DPP at 48 °C for 1 h, no detectable decrease in band intensity was observed. The band started to weaken from 51 °C, maintained until 57 °C, and finally disappeared when the temperature increased to above 60 °C (Figure 4B). These indicated that the presence of DPP significantly enhanced the thermal stability of OAT1 compared to the control group, conclusively demonstrating a direct binding interaction between DPP and OAT1. As nearly 86% of DPP was intact after incubation with 2 mM GSH at 37 °C for 1 h and then at 60 °C for 3 min (Figure S8A), which are the conditions similar to those for the thermal shift binding assays described above, the binding assays suggest that probenecid coordinated with Pt(IV) still retains binding affinity to OAT1.

### 2.7. Inhibition of OAT1 Expression

**Figure 5:**
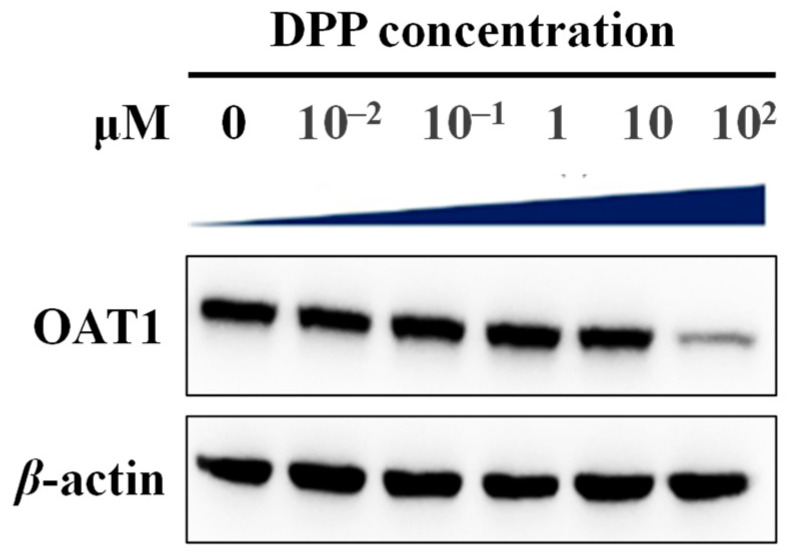
Expression of OAT1 protein in MCF-7 cells in the presence of different concentrations of DPP.

Given the inhibition ability of probenecid to OAT1 [34,35], and binding of DPP to OAT1 described above, we analyzed changes in intracellular OAT1 expression levels in MCF-7 cells after 24 h of treatment with various concentrations of DPP via Western blot assay. As shown in Figure 5, DPP showed little inhibitory effect on OAT1expression at low concentrations (0–10 μM), while significantly reducing the expression of OAT1 at a concentration of 100 μM. After 2 h of incubation with 2 mM GSH at 37 °C for 24 h, only one-third of DPP was reduced according to the UV–vis spectrum shown in Figure S8B. This implies that if 100 μM DPP is incubated in MCF-7 cells at 37 °C for 24 h, ~33 μM of CDDP-like species and ~66 μM probenecid will be released. These concentrations are much higher than the IC_50_ values of CDDP (5.29 μM; Table 1) against MCF-7 cancer cells and that of probenecid (12.3 μM [36]) for inhibiting OAT1 activity, respectively. These results suggest that the cytotoxicity of DPP is attributable to the released Pt(II) species, which mainly target nuclear DNA, and to the released/coordinated probenecid, which inhibits the activity of OAT1, but not to probenecid-mediated inhibition of OAT1 expression. 

## 3. Discussion

CDDP remains one of the most widely used anticancer agents for the treatment of multiple solid tumors; however, its severe side effects still limit its further clinical application, especially the dose-limiting damage to renal tubular cells [37]. The ability of CDDP to induce kidney damage was previously reported to depend on the uptake carrier organic cation transporter 2 (OCT2), which is highly expressed in renal proximal tubular cells, the cochlea, and dorsal root ganglia [38,39]. Later, an OAT1/OAT3-dependent pathway was also proposed [18]. A number of studies have reported that probenecid can reduce the tubular secretion of total platinum after CDDP administration in various animals [40,41,42] and humans [43], and act as a chemo-protector against CDDP-induced nephrotoxicity [44]. This inspires us to design and synthesize novel CDDP-based Pt(IV) anticancer prodrugs bearing axial probenecid ligands (SPP and DPP). Another key advantage of the probenecid ligand is its carboxyl group, which can be readily linked to the hydroxyl group of oxoplatin without additional modifications. Moreover, probenecid linking to the Pt center via its carboxyl group does not significantly affect its binding to OAT1, although the carboxyl group is partially involved in the recognition by OAT1, in addition to the phenyl ring, sulfonyl moiety, and two propyl groups, as revealed by cryo-EM structures [45,46,47]. 

The as-synthesized SPP and DPP prodrugs are stable in phosphate-buffered saline (PBS) but can be readily reduced in the presence of the cellular reducing agent GSH. Such stability can prevent their potential interactions with unwanted biomolecules in the bloodstream before entering cancer cells. Probenecid has also been reported to bind to plasma proteins with a high affinity, e.g., 85–95% binding to albumin [48], which might also facilitate the transportation of SPP and DPP. The significantly higher cellular uptake and the following promising cytotoxicity further confirmed this result. 

Although SPP and DPP exhibited promising anticancer activities against breast cancer cells, especially against the triple-negative breast cancer cell line MDA-MB-231, being more cytotoxic than CDDP, all three Pt complexes still induced a similar early apoptotic mechanism in breast cancer cells. This suggests that the probenecid introduced in SPP and DPP might not directly induce cancer cell death but instead synergistically acts on other cellular targets. This might be related to the versatile roles of probenecid in cells, such as anion transporter blockade [36], TRPV2 channel activation [49], Panx1 hemichannel blockade [23], modulation of multidrug resistance proteins (MRP) activity [22], and inhibition of glutathione conjugate export pumps [50]. The exact mechanism of action of such potential synergistic effects of probenecid ligands in SPP and DPP needs further exploration.

## 4. Materials and Methods

### 4.1. Materials

Cisplatin (CDDP) was purchased from Shanghai Maclin Biochemical Technology Co., Ltd. (Shanghai, China). DMEM basal medium was purchased from Gibco (USA). Probenecid was purchased from Shanghai Aladdin Biochemical Technology Co., Ltd. (Shanghai, China). O-(benzotriazole-1-yl)-N,N,N′,N′-tetramethylurea tetrafluoroborate (TBTU) was purchased from Shanghai Bide Pharmaceutical Technology Co., Ltd. (Shanghai, China). The Human breast cancer cell lines MCF-7 (CRL-3435), T47D (CRL-2865), and the triple-negative breast cancer cell line MDA-MB-231 (CRM-HTB-26) were obtained from the American Type Culture Collection (ATCC). Analytical-grade diethyl ether, ethanol, triethylamine (TEA), and N,N-dimethylformamide (DMF) were purchased from Sinopharm Chemical Reagent Co., Ltd. (Shanghai, China). Hydrogen peroxide (H_2_O_2_, 30%) was purchased from Tianjin Tianli Chemical Reagent Co., Ltd. (Tianjin, China). Silica gel powder, dichloromethane, and methanol were purchased from Wuhan Xinshenshi Chemical Technology Co., Ltd. (Wuhan, China). Other reagents are all analytical grade if not stated otherwise and used without further purification. Ultrapure water was used for all aqueous solutions.

### 4.2. Instruments

UV–vis spectra were recorded on a Shimadzu UV-3600 spectrophotometer (Shimadzu, Japan). High-resolution mass spectrometry (HR-MS) was performed on a Xevo G2 Q-TOF mass spectrometer (Waters, Manchester, UK). ^1^H and ^13^C NMR spectra were recorded on an Agilent 600 MHz DD2 spectrometer equipped with a single probe and z-field gradients (Agilent). Platinum content was quantified using an Agilent 7700x inductively coupled plasma mass spectrometry (ICP-MS) system (Agilent Technologies, Santa Clara, CA, USA). Flow cytometry was conducted on a BD FACSCalibur instrument (BD, USA). Cells were cultured in a SANYo CO_2_ incubator, and absorbance readings were taken on a Tecan Infinite F50 microplate reader (BD, Franklin Lakes, NJ, USA).

### 4.3. Synthesis of SPP

Probenecid (69.2 mg) was dissolved in DMF (5 mL), followed by the addition of TBTU (77.6 mg). After stirring for 15 min at room temperature, triethylamine (33 μL) was added. Then, after stirring for another 15 min, platinum hydroxide (66.6 mg) was added, and the reaction was maintained at 25 °C under N_2_ for 12 h. The mixture was precipitated by adding diethyl ether (200 mL) and stored at 4 °C overnight. The yellow precipitate was collected by centrifugation and purified by silica gel chromatography (CH_2_Cl_2_:CH_3_OH = 5:1), and then dried in vacuo to afford a pale-yellow solid (yield: 21%). ^1^H NMR (600 MHz, DMSO-*d*_6_) δ(ppm) 8.01 (d, *J* = 8.0 Hz, 1H), 7.82 (d, *J* = 8.1 Hz, 1H), 6.29–5.83 (m, 3H), 3.01 (t, *J* = 7.6 Hz, 2H), 1.45–1.38 (m, 2H), 0.78 (t, *J* = 7.3 Hz, 3H). ^13^C NMR (600 MHz, DMSO-*d*_6_) δ(ppm) 177.08, 146.77, 143.58, 135.27, 131.58, 54.58, 26.57, 16.18. ESI-MS (+): C_13_H_25_Cl_2_N_3_O_5_PtS, calculated *m*/*z* 602.06, found *m*/*z* 602.06.

### 4.4. Synthesis of DPP

Probenecid (171.216 mg) was dissolved in DMF (20 mL), followed by the addition of TBTU (192 mg). After stirring for 15 min at room temperature, triethylamine (86 μL) was added. Then, stirring was continued for another 20 min, platinum hydroxide (66.6 mg) was added, and the reaction was maintained at 55 °C under N_2_ for 24 h. The mixture was precipitated with diethyl ether (200 mL) and stored at 4 °C overnight. The yellow precipitate was collected by centrifugation, washed with methanol and ether, and purified by silica gel chromatography (CH_2_Cl_2_:CH_3_OH = 5:1), and then dried in vacuo to afford a pale-yellow solid (yield: 51%). ^1^H NMR (600 MHz, DMSO-*d*_6_) δ (ppm) 8.06 (d, *J* = 8.2 Hz, 1H), 7.88 (d, *J* = 8.3 Hz, 1H), 6.77 (s, 1.5H), 3.04 (t, *J* = 7.5 Hz, 2H), 1.45 (m, *J* = 7.4 Hz, 2H), 0.80 (t, *J* = 7.3 Hz, 3H). ^13^C NMR (600 MHz, DMSO-*d*_6_) δ(ppm) 172.11, 142.55, 136.77, 130.64, 126.97, 49.83, 21.84, 11.43. ESI-MS (+): C_26_H_42_Cl_2_N_4_O_8_PtS_2_, calculated *m*/*z* 868.74, found *m*/*z* 868.19.

### 4.5. Stability and Reduction of Pt Complexes

SPP and DPP (20 μM each) were dissolved in PBS containing 2% DMSO in the absence or presence of 200 μM glutathione (GSH). The solution was incubated at room temperature, and aliquots were taken at various time points to be monitored by a UV–vis spectrophotometer in the range of 200 to 400 nm.

### 4.6. Antiproliferative Activity

All cells were cultured at 37 °C in a humidified atmosphere of 5% CO_2_ and allowed to adhere for 24 h. MCF-7, MDA-MB-231, and T47D cells were digested with trypsin and seeded in 96-well plates at a density of 8–10 × 10^4^ cells/well. Different concentrations (0.1–100 μM) of the tested compounds were added to each well of the 96-well plate, respectively. CDDP was applied as a positive control. The plate was incubated for 72 h, and the cell viability was determined using the standard CCK-8 assay in three independent experiments.

### 4.7. Cellular Uptake of Pt Complexes

MCF-7 cells were incubated with 20 μM DPP, SPP, or CDDP for 24 h at 37 °C for 24 h. The cells were then washed three times with ice-cold PBS to remove uninternalized drugs and subsequently digested with trypsin. Cell density was determined using an automatic cell counter (Luna-II, Logos Biosystems, Gyeonggi-do, Republic of Korea). The cell pellets were digested with 1% nitric acid (HNO_3_, TraceSELECT^®^ Ultra, Sigma-Aldrich, Shanghai). Platinum content was quantified by inductively coupled plasma mass spectrometer (ICP-MS, Agilent 7700x, Agilent Technologies, Santa Clara, CA, USA). The typical parameters were as follows: RF power 1550 W, carrier gas flow rate 1.05 L/min, sampling depth 8.0 mm, and data were collected in standard mode. A multi-element calibration standard solution (Agilent Technologies) was used to construct a calibration curve, with ^193^Ir selected as the internal standard for quantitative correction. The experimental results are expressed as the weight of platinum (ng) in every 10^6^ cells, and the platinum content in whole cells and nuclei was determined separately. All experiments were repeated three times independently, and the data were presented in the form of mean ± standard deviation (SD).

### 4.8. Apoptosis Assay

MCF-7 cells were seeded in a 6-well plate at a density of 5 × 10^5^ cells/well and allowed to adhere for 24 h. Then, the MCF-7 cells were incubated with 20 μM CDDP (positive control), 20 μM SPP, or 20 μM DPP for 24 h, respectively. After incubation, the cells were washed, trypsinized, and centrifuged. After washing the cells with PBS, the cells were resuspended with 100 μL of 1 × Binding Buffer, stained with 5 μL of Annexin V-FITC and 5 μL of propidium iodide (PI) (50 μg/mL) for 15 min in the dark. Stained cells were analyzed by a BD FACSCalibur flow cytometer, and FL1 (530/30 nm) and FL2 (585/42 nm) channels were set to detect FITC and PI fluorescence signals, respectively. Finally, the data were analyzed using FlowJo v10.8.1 software. All experiments were repeated three times independently.

### 4.9. Western Blot Analyses

MCF-7 cells were treated with DPP in different concentrations (0.01, 0.1, 1, 10, 100 μM) for 24 h. The proteins from the cell lysates were electrophoretically separated on 8% SDS-PAGE gels. The proteins were transferred onto a polyvinylidene fluoride (PVDF) membrane and probed with an anti-OAT1 antibody (1:1000, CST) and a mouse anti-β-actin antibody (1:5000, Bioprimacy Biology Technology Co., Ltd. (Wuhan, China)). After washing the membrane with TBS containing 0.1% Tween-20, the second antibodies of sheep anti-rabbit IgG (1:6000, Wuhan Kerui Biotechnology Co., Ltd. (Wuhan, China)) and sheep anti-mouse IgG (1:6000, Wuhan Kerui Biotechnology Co., Ltd. (Wuhan, China)) were added. The membrane was washed with TBS containing 0.1% Tween-20 and tested by enhanced chemiluminescence (ECL). The bands were quantitatively analyzed using the ImageJ software (version 1.51j8). 

### 4.10. Thermal Shift Assay

MCF-7 cells were subjected to seven consecutive freeze–thaw cycles in liquid nitrogen to facilitate cell lysis. Subsequently, protein concentrations in these lysates were determined using the BCA assay. The resulting cell lysates, standardized to 1 mg/mL, were divided into two groups: one treated with DMSO (vehicle control) and the other with 100 μM of DPP, followed by incubation at 37 °C for 1 h. The samples were then heated at temperatures ranging from 25 to 66 °C for 3 min, followed by a 3 min incubation at room temperature. The samples were centrifuged at 20,000× *g* for 20 min to separate protein aggregates from soluble proteins. The resulting supernatants were analyzed by Western blotting.

## 5. Conclusions

In this study, we designed and synthesized two Pt(IV) anticancer prodrugs, SPP and DPP, with mono- and di-probenecid at the axial positions, respectively. Both complexes exhibited significant antiproliferative activity against human breast cancer cell lines, and they were particularly cytotoxic to TNBC cells. The stability before reaching cancer cells, ready reduction by GSH, and enhanced cellular uptake are proposed to contribute to their promising anticancer activity. The results herein indicate the advantages of carboxyl-containing old drugs in constructing a novel CDDP-based Pt(IV) prodrug, and it is a convenient and effective method to improve the metabolic actions and enhance the cytotoxicity of CDDP. Considering the versatile potential medicinal roles of the probenecid, e.g., in the psychiatric field due to its ability to pass through the blood–brain barrier [16,17,48], the exact mechanism of action still needs further exploration, which might bring great improvements to the chemotherapy of refractory tumors by platinum drugs.

## Figures and Tables

**Table 1:** IC_50_ values (μM) of SPP and DPP against different breast cancer cell lines with CDDP as a positive control.

Pt Complexes	IC_50_ Mean ± SD (μM)		
	MCF-7	T47D	MDA-MB-231
SPP	4.13 ± 0.11	4.06 ± 0.09	1.94 ± 0.15
DPP	0.61 ± 0.02	0.52 ± 0.04	1.78 ± 0.05
CDDP	5.29 ± 0.15	3.77 ± 0.04	4.12 ± 0.17

## Data Availability

The original contributions presented in this study are included in the article/Supplementary Materials. Further inquiries can be directed to the corresponding authors.

## Supplementary Materials

The following supporting information can be downloaded at: https://www.mdpi.com/article/10.3390/ph19030386/s1, Figure S1: Mass spectrum of SPP; Figure S2: Mass spectrum of DPP; Figure S3: ^1^H NMR spectrum of SPP; Figure S4: ^1^H NMR spectrum of DPP; Figure S5: ^13^C NMR spectrum of SPP; Figure S6: ^13^C NMR spectrum of DPP; Figure S7: Anti-proliferative activities; Figure S8: UV–vis spectrum of DPP in the presence of 2 mM GSH.
