# Supplementary material for: Multitargeting Pt(IV) Anticancer Prodrugs Bearing Mono- and Bis-Probenecid Ligands in Axial Positions: Synthesis and Evaluation of Biological Activity
Source: Pharmaceuticals (Basel). 2026 Feb 27;19(3):386. doi: 10.3390/ph19030386 (PMC13029561; doi:10.3390/ph19030386)
Supplement: Supplementary file 1 [file pharmaceuticals-19-00386-s001.zip › pharmaceuticals-4154797-supplementary.pdf]

Supplementary Materials: Multitargeting Pt(IV) Anticancer Prodrugs Bearing Mono- and Bis-Probenecid Ligands in Axial Positions: Synthesis and Evaluation of Biological Activity

Panxing Qiu, Yu Zhang, Yang Dou, Zhijin Cheng, Xiaoqin Wu, Silong Zhang, Fuyi Wang and Kui Wu

Figure S1. Mass spectrum of SPP.

Figure S2. Mass spectrum of DPP.

Figure S3. ^{1}H NMR spectrum of SPP.

Figure S4. ^{1}H NMR spectrum of DPP.

Figure S5. ^{13}C NMR spectrum of SPP.

Figure S6. ^{13}C NMR spectrum of DPP.

Figure S7. Anti-proliferative activities.

Figure S8. UV-Vis spectrum of DPP in the presence of 2 mM GSH.

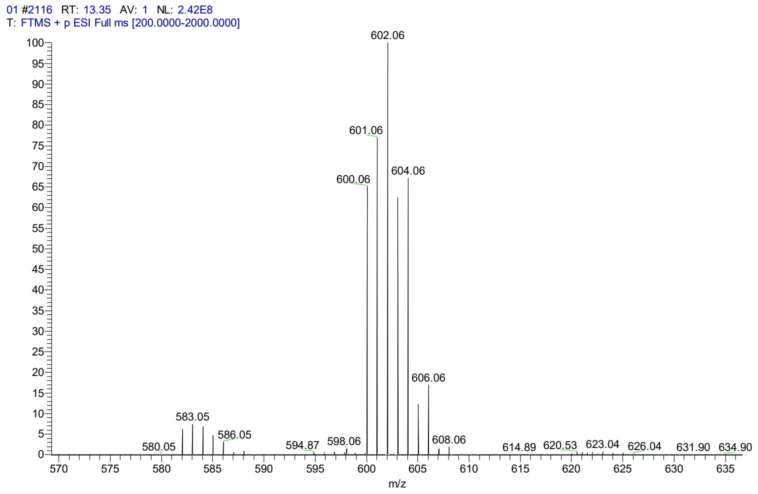

Figure S1. Mass spectrum of SPP.

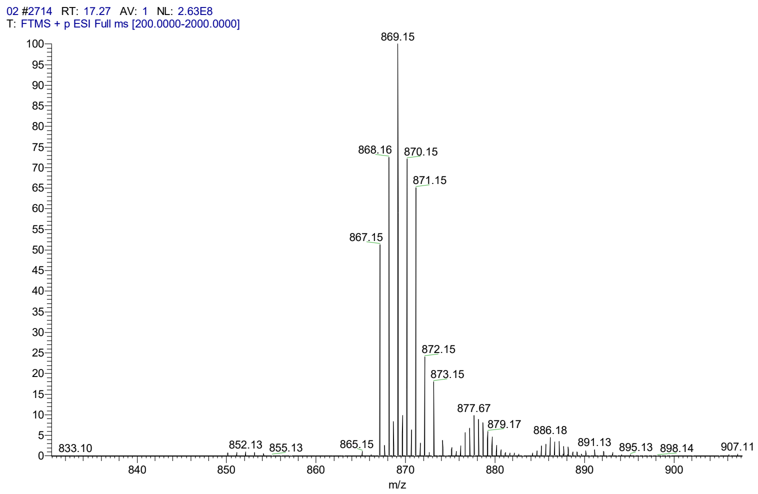

Figure S2. Mass spectrum of DPP.

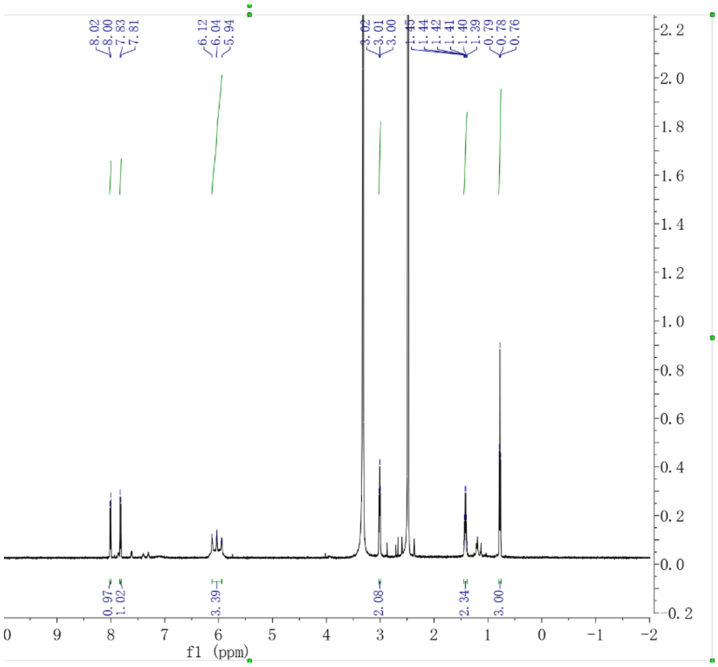

Figure S3. ^{1}H NMR spectrum of SPP.

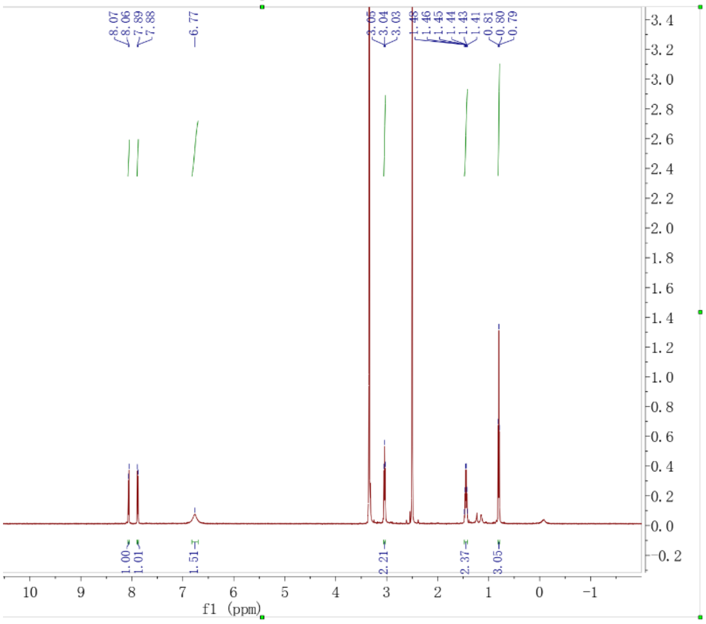

Figure S4. ^{1}H NMR spectrum of DPP.

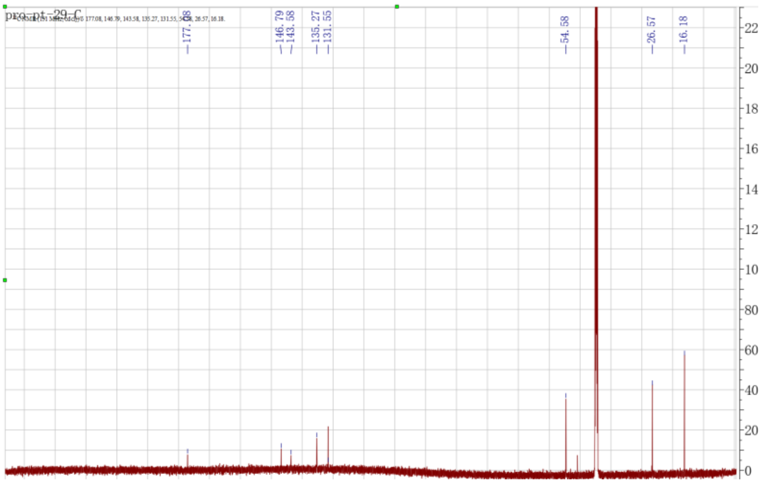

Figure S5. ^{13}C NMR spectrum of SPP.

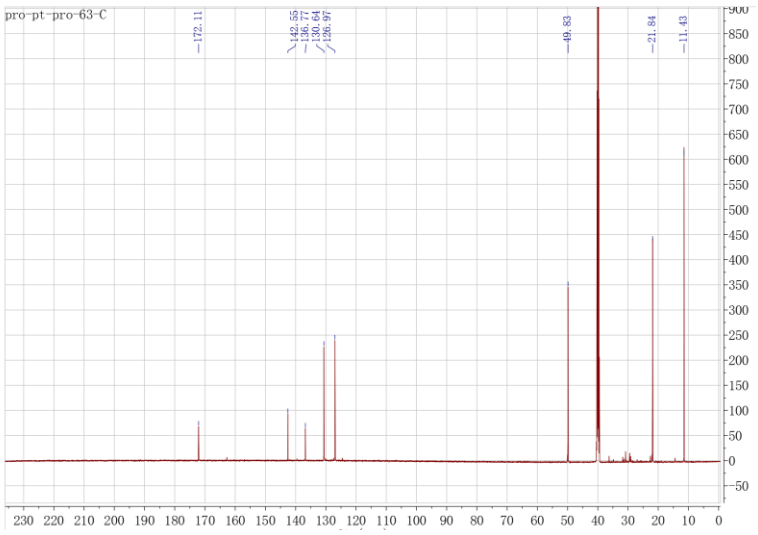

Figure S6. ^{13}C NMR spectrum of DPP.

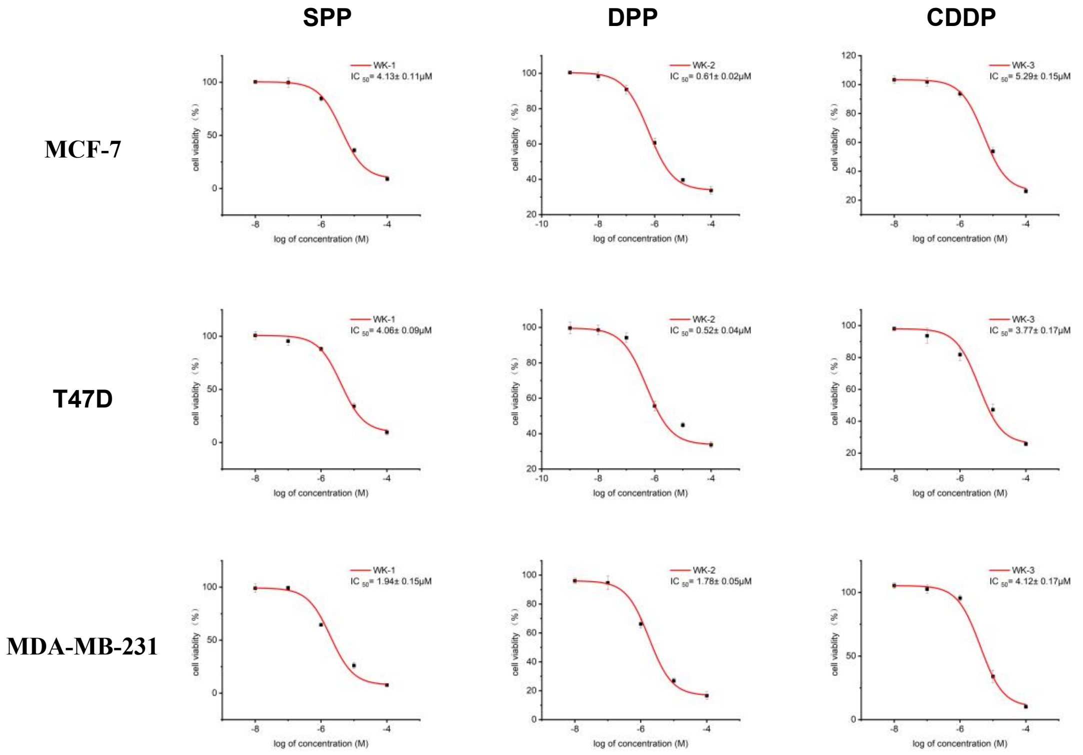

Figure S7. Inhibition curves of SPP, DPP and CDDP on the proliferation of MCF-7, T47D and MDA-MB-231 breast cancer cells.

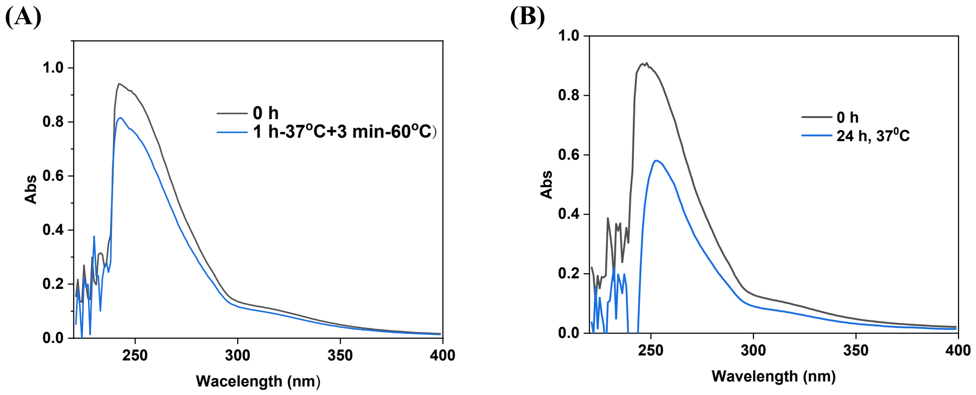

Figure S8. UV-Vis spectra of DPP in the presence of 2 mM GSH under the same conditions as those for the thermal binding shift assay (A) and the inhibition assay (B).
